# Embolism formation and repair of *Phyllostachys vivax* f. *aureocaulis* in winter and the role of non-structural carbohydrates in this process

**DOI:** 10.3389/fpls.2025.1539320

**Published:** 2025-05-29

**Authors:** Juan Li, Jinge Wang, Lei Chen, Chongyang Wu, He Li, Zhanchao Cheng, Jian Gao

**Affiliations:** ^1^ Key Laboratory of Bamboo and Rattan Science and Technology, International Centre for Bamboo and Rattan, Beijing, China; ^2^ Horticulture Center, Beijing Botanical Garden Management Office, Beijing, China

**Keywords:** bamboo, freeze-thaw embolization, hydraulic conductivity, non-structural carbohydrates, anatomical structure, reparation

## Abstract

*Phyllostachys vivax* f*. aureocaulis* is an important ornamental plant in Beijing and is primarily cultivated in the Henan, Zhejiang, and Jiangsu provinces of China. Low winter temperatures can lead to xylem embolism and plant mortality; however, there is a scarcity of studies focused on bamboo. Abnormal climatic conditions in Beijing frequently occur during winter, making it essential to investigate changes in the embolism and recovery processes of bamboo in this region. This experiment was conducted from December 2021 to March 2022 to measure the embolization curve and variations in embolization sites across different organs. This study also examined the relationship between hydraulic structural characteristics and embolization regulation by integrating physiological water indices and anatomical modifications. The results revealed that the embolization ratios of the vascular conduits in the culm, twig, and petiole ranged from 99.44% to 54%, 99% to 67%, and 99% to 57%, respectively, from December to March. Vulnerability to culm embolization was greater in January and February than in December and March, with the highest vulnerability observed in February. Non-structural carbohydrates (NSCs) in rhizomes located 15 and 30 cm distance from the culm were significantly higher in March than in December (*P<* 0.05), accompanied by a significant increase in starch content. The NSC and starch contents in the 10th and 15th culm internodes were significantly elevated in March compared with those in January and February. The net photosynthetic rate was higher in December and March, lower in January and February, and more negative in February. The diameters of the short and long axes of the pit in the duct, the number of parenchyma cell pits, and the area of parenchyma cell pits were significantly reduced in January and February compared with those in December and March (*P*< 0.05). In December and January, the vascular bundles and cells appeared intact; however, in February, signs of fragmentation and invasion by the filling bodies were observed. By March, the vascular bundles and cells had returned to normal, with a substantial presence of starch granules in the parenchyma cells. These findings provide a basis for introducing bamboo species that can withstand climate change and abnormal winter conditions as well as for implementing effective scientific management strategies.

## 1 Introduction

Over the past 30 years, winter temperatures in eastern China, particularly Beijing, have increased significantly. However, abnormal regional cold events have also occurred owing to global climate change (Ding, 2008; Easterling and Wehner, 2009; Zhu et al., 2016). Warming and shortening of winters have reduced the damage to introduced bamboo, and the duration of xylem embolism in bamboo has decreased. Nevertheless, extreme and repeated low temperatures can lead to freezing and thawing of bamboo xylem. Investigating the mechanisms underlying the limitations of water transport caused by xylem embolism and the depletion of non-structural carbohydrates (NSCs) in introduced bamboo species that endure winter is of great significance (Sala et al., 2010; McDowell, 2011).

Increased xylem tension caused by cold and drought stress, as well as a sudden phase transition from liquid water to gaseous water vapor, leads to embolism. This results in a loss of hydraulic conductivity in plants and a reduction in water transport to the canopy, thereby affecting a range of physiological activities (Choat et al., 2018). Freeze-thaw embolization primarily occurs during multiple freeze-thaw cycles in the xylem (Charra-Vaskou et al., 2016). After undergoing several freeze-thaw cycles, the xylem becomes increasingly sensitive to hydraulic loss (Dai et al., 2020). In addition, damage to the cell wall of the ducts and the porous membrane significantly diminishes the ability of plants to withstand subsequent freeze-thaw embolism, leading to a phenomenon known as “freeze fatigue.” After freezing and thawing, many tree species can almost completely lose their hydraulic conductivity throughout winter (Christensen-Dalsgaard and Tyree, 2014).

NSCs are primarily composed of soluble sugars and starches that contribute to carbon (C) supply. They are distributed and stored in parenchyma cells (Hartmann and Trumbore, 2016; Hartmann et al., 2020). Under stress conditions, water transport and C dynamics are interconnected (Sala et al., 2010; McDowell, 2011; Sevanto et al., 2014). The dynamics of NSCs among plant organs reflect the status of C sources and sinks as well as the functions of these organs in adapting to environmental changes (Hartmann and Trumbore, 2016). Recent findings suggest that NSCs may play a crucial role in helping plants survive harsh conditions (O’Brien et al., 2014; Blumstein et al., 2020). They contribute to the maintenance of hydraulic integrity by facilitating cell maintenance and osmotic regulation (Sala et al., 2012; O’Brien et al., 2014; Sevanto et al., 2014; Secchi et al., 2017). When NSC levels fall below a critical threshold, plant metabolism is severely restricted, leading to a hydraulic imbalance and hindering the repositioning and utilization of NSCs (Barotto et al., 2016). The larger the diameter of the pitted pores in the ducts, the greater the permeability of the pitted membranes, which increases the likelihood of xylem embolism (Tyree and Ewers, 1991). Species with larger bundle diameters, thicker bundle walls, richer axial thin walls, and higher ratios of palisade-to-spongy tissue thickness exhibit a greater capacity for NSC storage (Li et al., 2022), aiming to balance water transport efficiency and safety against the potential risk of embolization (Hacke et al., 2001). However, the role of NSCs in bamboo plants during winter and the mechanisms related to embolism mitigation remains unclear.

Bamboo is a type of perennial monocotyledonous plant. Unlike trees, bamboo does not exhibit secondary culm growth and is therefore unable to produce new xylem. Consequently, long-distance water transport must be restored by repairing the freeze-thaw embolisms. Vascular bundles in bamboo leaves and culms undergo a daily cycle of cavitation, embolization, and hydration (Holloway-Phillips and Brodribb, 2011; Yang et al., 2012). Therefore, studying winter embolization recovery in bamboo can provide valuable insights into the repair mechanisms of freeze-thaw embolisms. Bamboo demonstrates low water transport safety owing to the presence of vascular bundles in many species with diameters of >40 μm. These bundles are susceptible to cavity formation during freezing and thawing, resulting in the creation of numerous small bubbles that intermingle with liquid water during the thawing process, ultimately leading to embolisms (Sha et al., 2009). Consequently, bamboo xylose ducts are highly sensitive to adverse effects (Sha et al., 2009). However, the mechanisms by which hydraulic structures regulate embolization at low temperatures remain unclear.

*Phyllostachys vivax* f. *aureocaulis* belongs to the *Bambusoideae* subfamily of the *Gramineae* family. It exhibits a monopodial and scattered growth pattern and represents a variant of *P. vivax* McClure. This species is highly ornamental, serves as a valuable resource for urban greening initiatives, and is primarily distributed in Jiangsu, Zhejiang, and other regions of China. Since the 1980s, a significant number of *P. vivax* f. *aureocaulis* have been introduced to regions such as Beijing, Shandong, Shaanxi, and Henan. Various researchers have investigated the biological and physiological parameters of these introduced species and have observed that *P. vivax* f. *aureocaulis* can successfully survive after introduction. In this study, *P. vivax* f. *aureocaulis* was selected as the experimental plant to examine its water conductivity loss rate (PLC) and changes in embolism across different tissues during the winter. In addition, this study investigated the repair mechanisms involved in the mutual conversion of NSCs and photosynthesis. Analysis of the xylem conduit and pit structure revealed embolic changes and NSC repair mechanisms in *P. vivax* f. *aureocaulis*. Two questions were addressed: What is the relationship between the dynamic changes in NSC components and embolisms? What is the relationship between the culm vulnerability and its microscopic structure during winter, as well as its potential connection to NSCs? These findings provide a foundation for introducing bamboo species that can adapt to climate change and abnormal winter conditions as well as for implementing scientific management strategies.

## 2 Materials and methods

### 2.1 Plot setup and sample collection

Beijing experiences a typical warm temperate semi-humid continental monsoon climate, characterized by hot, rainy summers and cold, dry winters with wind and minimal snowfall. Extreme minimum winter temperatures generally range from -14°C to -20°C. This experiment was conducted in a *P. vivax* f. *aureocaulis* forest at the China National Botanical Garden (North Garden) in Beijing. Three plots measuring 10 × 10 m^2^ were established for the study, which was conducted from December 2021 to March 2022. The illumination (lux), air temperature (°C), and relative humidity (%) were continuously monitored and recorded using a microclimate meter (Pocket Weather Tracker 4000). The vapor pressure deficit (VPD) was calculated based on the relative humidity (RH) and air temperature (Ta). The average plant height was between 8 and 10 m, and the diameter at breast height was between 7 and 8 cm.

New leaves from 20 bamboo plants, each measuring 8–10 m in height and having a ground diameter of 4–6 cm, were selected for photosynthesis determination. Three plants were sampled each month to measure photosynthetic parameters. Ten-millimeter segments were collected from the 5th, 10th, 15th, and 20th internodes of the culm samples and immersed in FAA (formaldehyde: acetic acid: ethanol = 5%:5%:90%) fixation solution. Half of the samples were analyzed using scanning electron microscopy (SEM), whereas the other half were assessed for NSCs one week later. The 10th culm segment was selected, and a 10 cm culm, 3 cm primary twig, and 1 cm petiole were collected. After sampling, both ends of each sample were immediately sealed with hot-melt glue, placed in an icebox, and transported to the computed tomography (CT) room for scanning. Ten to 15 culm and rhizome segments from the 15th to 20th internodes were selected for conductivity loss rate determination, with each segment measuring 10–13 cm in length.

### 2.2 Micro-CT scans and image reconstruction

After sampling, both ends of each sample were immediately sealed with hot-melt glue, placed in an icebox, and sent to a CT room for scanning. The Skyscan 1172 micro-CT scanner used in this study (Bruker Corporation, Kontich, Belgium) has a significantly lower inlet dose rate (<1 mGy s^-1^) than the previously reported dose rate for micro-CT scans at the beamline of synchrotron radiation facilities (47 mGy s^-1^) (Petruzzellis et al., 2018).

Based on preliminary experiments, the scanning parameters were adjusted to minimize the ionizing radiation dose while maintaining an adequate contrast and resolution. The final scanning parameters were as follows: source voltage, 49 keV; source current, 200 μA; exposure time, 220 ms; rotation step, 0°–180° at 0.4° intervals; image pixel size, 3.92 μm, and total scanning time, 15 minutes. The length of each branch was measured at 5.2 mm, and 499 two-dimensional projection images, each with a resolution of 2000 × 1332 pixels, were obtained.

After each micro-CT scan, 499 X-ray projections were obtained and reconstructed using the NRecon software (Bruker Corporation, Kontich, Belgium) to create three-dimensional (3D) images. For each branch segment, the spatial structure of the xylem was aligned with a series of 3D images obtained from repeated scans using the “co-registration” function in Data Viewer software (Bruker Corporation, Kontich, Belgium). A synchronized 3D image with a longitudinal extent of 1 mm (comprising 257 cross-sectional images) was selected, positioned 2 mm below the short line mark in each organ segment, and converted to eight bits using CT An software. Images were processed using ImageJ software (Bruker Corporation, Kontich, Belgium). The tubes and vascular bundles were isolated with water, and a distinction was made between the tubes and those filled with water from the embolized vascular bundles.

The embolization ratio was calculated using the following formula:


Embolization ratio=number of embolized vessels/total number of vessels

### 2.3 Hydraulic conductivity and PLC analysis

Ten to 15 culms from the 15th to 20th segments, including internodes, nodes, and five rhizome segments from the three bamboo samples, were washed to remove the soil and subsequently cut in deionized water. Samples were collected before dawn to assess water conductivity. The samples were then placed in a 0.1% potassium chloride solution, wrapped in a black plastic bag, and promptly transported to the laboratory to measure the actual water hydraulic conductivity. The hydraulic conductivity of the culm and rhizome segments was measured under a specified pressure.

**Figure 1:**
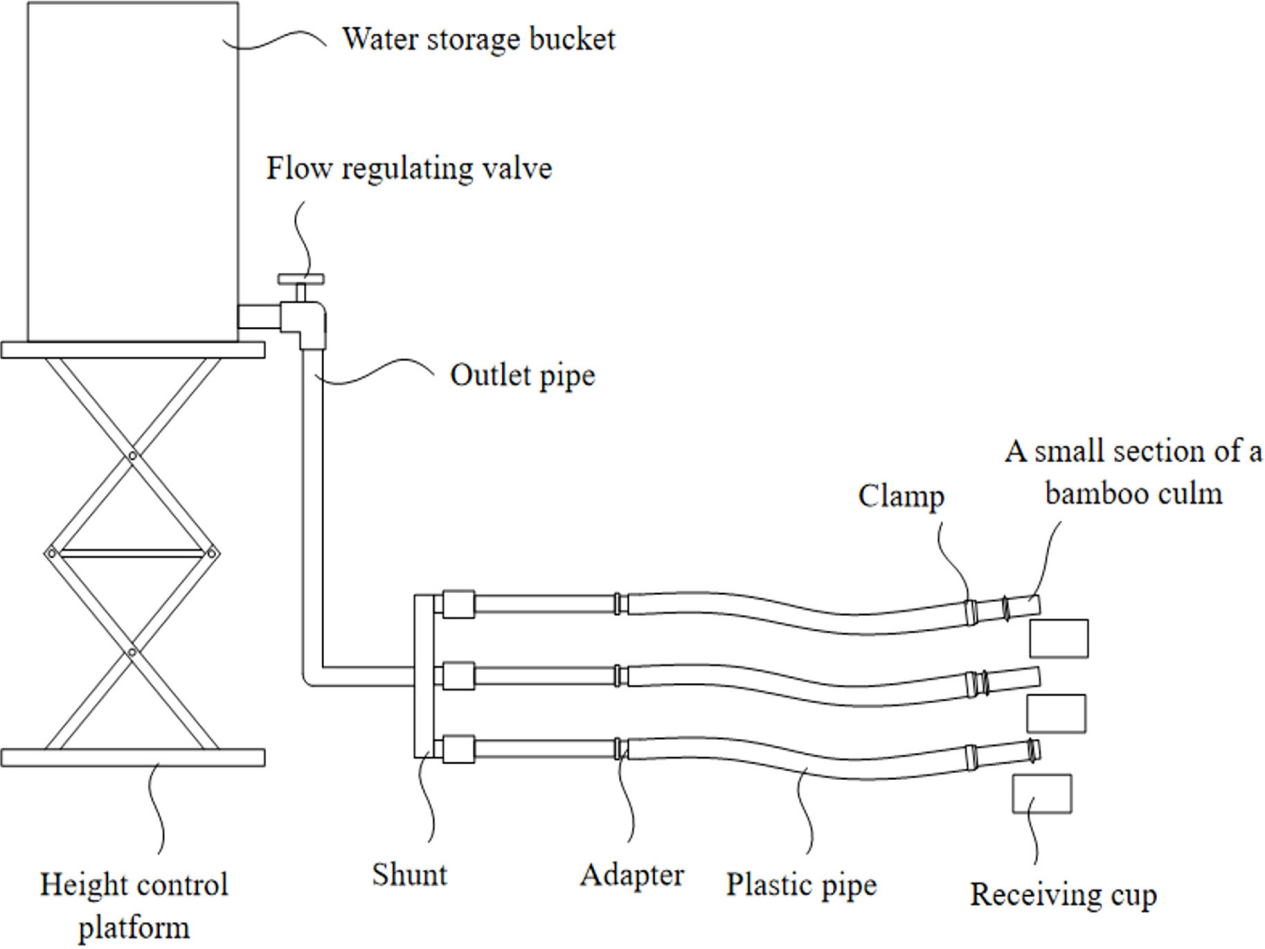
Structure diagram of measuring device for water conductance of bamboo culm.

The design of the bamboo culm water-conductivity device is illustrated in **Figure 1**. Multiple bamboo culms, each containing nodes, were connected using hoses and pipe clamps were used to fix the hoses to the bamboo culms, thereby preventing water leakage at the connection points. A potassium chloride solution was poured into a water storage container to a height of 1.3 to 1.7 meters, thereby establishing a pressure of 1.3 to 1.7 kilopascals within the container. By opening the flow control valve, the potassium chloride solution flowed through the outlet pipe and was directed toward the end of the bamboo culm. At this juncture, the water potential at the end of the bamboo culm initiated the movement of water through the culm.

The other end of the culm was then placed in a water-receiving container. The actual water conductivity of the bamboo culm was determined by measuring the water intake in the container.


$$K_{\text{x}}=\frac{QL}{A_{\text{x}}\Delta h}$$

where *K*_x_ is the xylem hydraulic conductivity coefficient (kg·m^−1^·s^−1^·MPa^−1^), *A*_x_ is the cross-sectional area (m^2^), Δh is the water potential pressure (Mpa^−1^), Q is the mass of aqueous solution flowing out per second (kg·s^-1^) and L is the stalk length (m) of the sample. High pressure ranges from 0.13 to 0.15 Mpa whereas low pressure ranges from 0.03 to 0.05 Mpa.

The highest and lowest water conductivities of all samples were measured using a self-guided water conductivity device. The samples were then placed in 10 ml centrifuge tubes and centrifuged (Luxiangyi High-Speed Centrifuge, Shanghai, China) for 2 minutes at a rotational speed of 300 RPM. The water conductivity (**Figure 1**) and water potential (PSΨPRO Water Potential Measurement System, Hansha Scientific Instrument, England) of the samples were determined by centrifugation. The samples were centrifuged 15–20 times to obtain the water conductivity gradient and the corresponding water potential.

PLC values at a corresponding tension were calculated as 100 × (K_max_ −K_h_)/K_max_. The Fitplc package in R was used to analyze the effect of plant leaf water potential on the PLC. The Weibull model was used to fit all data, and bootstrapping was conducted 50 times to estimate the uncertainty of the parameters. Vulnerability curves (VCs) generated by these methods were used to compare the percentage loss of conductivity of bamboo under varying water potentials.

The calculation formula is as follows:


$$\frac{\text{PLC}}{100}=1-\text{exp}\left[-{\left(\frac{\text{T}}{\text{b}}\right)}^{\text{c}}\right]$$

where b and c are parameters and T is the xylem tension (equal to negative xylem water potential).

The VCs of *P. vivax* f. *aureocaulis* were fitted using the dual-Weibull equation.


$$\text{PLC}=\text{β}\left\{1-\text{exp}\left[-{\left(\text{T}/{\text{b}}_{1}\right)}^{{\text{c}}_{1}}\right]\right\}+\left(100-\text{β}\right)\{1-\text{exp}\left[-{\left(\text{T}/{\text{b}}_{2}\right)}^{{\text{c}}_{2}}\right]\}\;=\;W1+W2$$

where W1 and W2 represent the two Weibull curves, β denotes the maximum PLC for W1, whereas b_1_ and c_1_ are constants for W1, and b_2_ and c_2_ are constants for W2. The xylem pressure corresponding to 50% PLC (P50), 12% PLC (P12), and 88% PLC (P88) was calculated.

### 2.4 Determination of NSCs

The extraction and determination of NSCs was performed according to the method described by Zhong et al. (2013). Approximately 0.0100 g of crushed dry sample was weighed and placed in a test tube. Subsequently, 3 mL of distilled water was added and the mixture was incubated in a water bath at 80°C for 30 minutes. After cooling, the samples were centrifuged at 10,000 g for 10 minutes. The resulting supernatant was used to determine the soluble sugars. A 200 µL aliquot of the supernatant was mixed with 2 mL of 0.4% anthrone-concentrated sulfuric acid. The mixture was thoroughly shaken, placed in a boiling water bath for 5 minutes, cooled to room temperature, and the absorbance was measured at 640 nm. A standard curve was constructed using sucrose.

### 2.5 Determination of photosynthetic parameters

For photosynthetic measurements, nine healthy leaves were selected from each bamboo plant, with one leaf taken from each cardinal direction at chest height. The net photosynthetic rate (*P*_n_) and transpiration rate (*T*_r_) were measured at three-time intervals, 9:00–10:00 AM, 12:00–1:00 PM, and 3:00–4:00 PM, representing the early, middle, and late parts of the day, respectively. Measurements were conducted using a photosynthesis determination system (Li-COR 6400, USA). The light intensity, CO_2_ concentration, and temperature were maintained at 800 mol/m^2^/s, 400 μmol/mol, and 25°C, respectively.

### 2.6 Scanning electron microscope (SEM)

**Figure 2:**
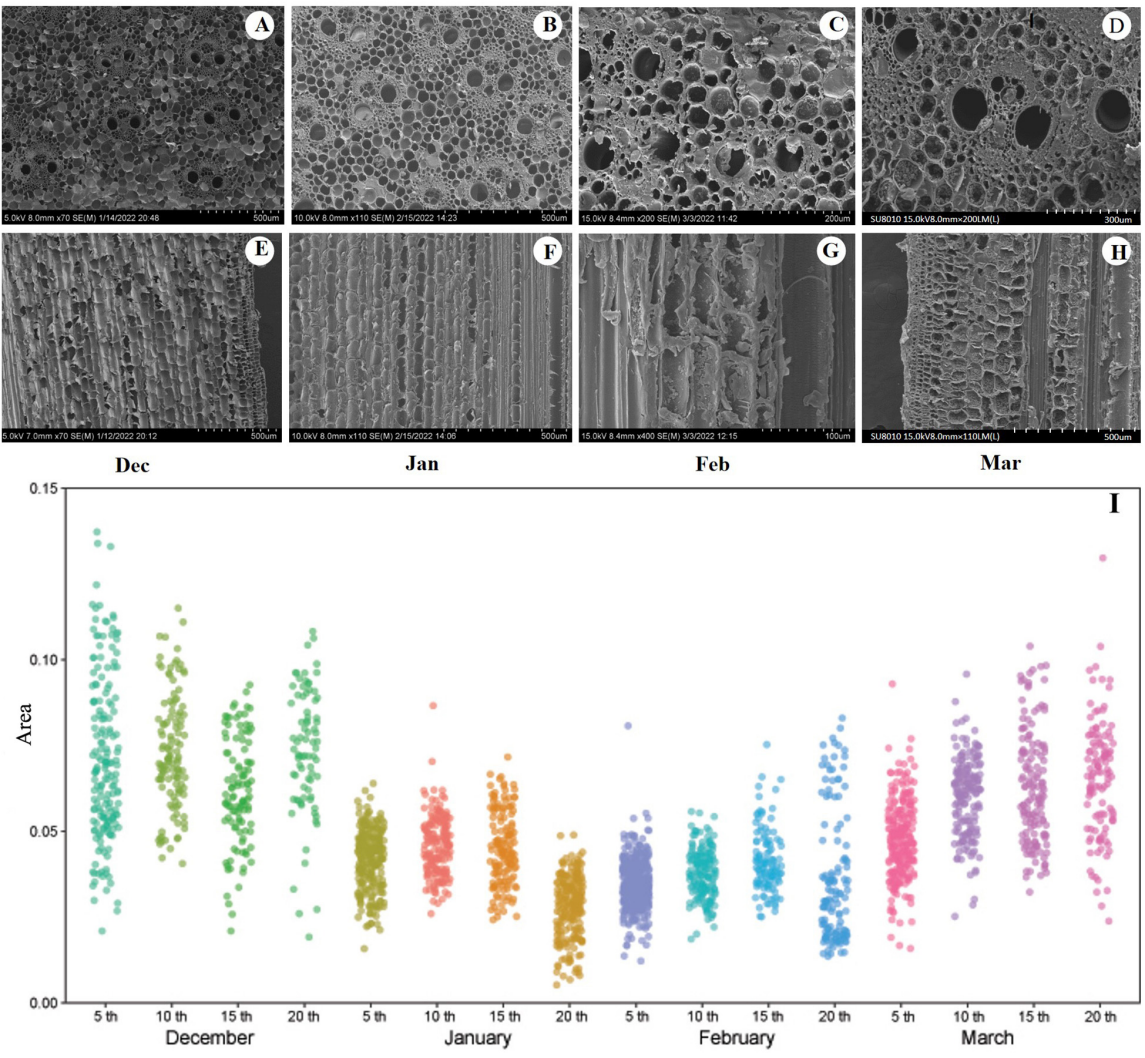
**(A–D)** Cross section of the tenth section of the bamboo culm in December, January, February and March respectively **(E–H)** Longitudinal section of the tenth section of the bamboo culm of the bamboo in December, January, February, March respectively. **(I)** Changes of vascular bundle area in different segments of bamboo culm during the overwintering period, n=6, three repetitions of each experiment. 5th,10th,15th,20th represent the fifth, 10th, 15th, and 20th culm section from the base up, respectively. In **(A, E)** n=12, three repetitions of each experiment, a total of 78 pictures; in **(B, F)** n=12, three repetitions of each experiment, a total of 72 pictures; in **(C, G)** n=12, three repetitions of each experiment, a total of 68pictures; in **(D, H)** n=12, three repetitions of each experiment, a total of 73 pictures.

Culm samples measuring 5, 10, 15, and 20 mm were collected and immersed in the FAA fixation solution. The area of the vascular bundles, the thickness of the parenchyma cells, pitted areas of the conduits and parenchyma cells, and the diameter of the pitted membranes were observed using SEM. The shooting face of each sample was affixed to a conductive adhesive and impurities on the sample surface were removed using an earbud. The samples were then placed in the equipment for gold sputtering, which lasted 90 seconds. Once the gold sputtering was completed, the sample stage was removed and placed in the SEM equipment for vacuum extraction. After vacuum extraction, imaging was performed using a Hitachi SU8010 instrument at 15 kV. Multiple images are presented in **Figures 2A–H**.

### 2.7 Statistical analyses

Data are presented as the mean ± standard deviation. Statistical analyses were conducted using SPSS version 26.0 for one-way analysis of variance (ANOVA), least significant difference tests, and multiple comparisons (α = 0.05). SPSS version 26.0 and Excel 2016 were used to create linear models and correlation graphs.

## 3 Results

### 3.1 Microclimate change

**Figure 3:**
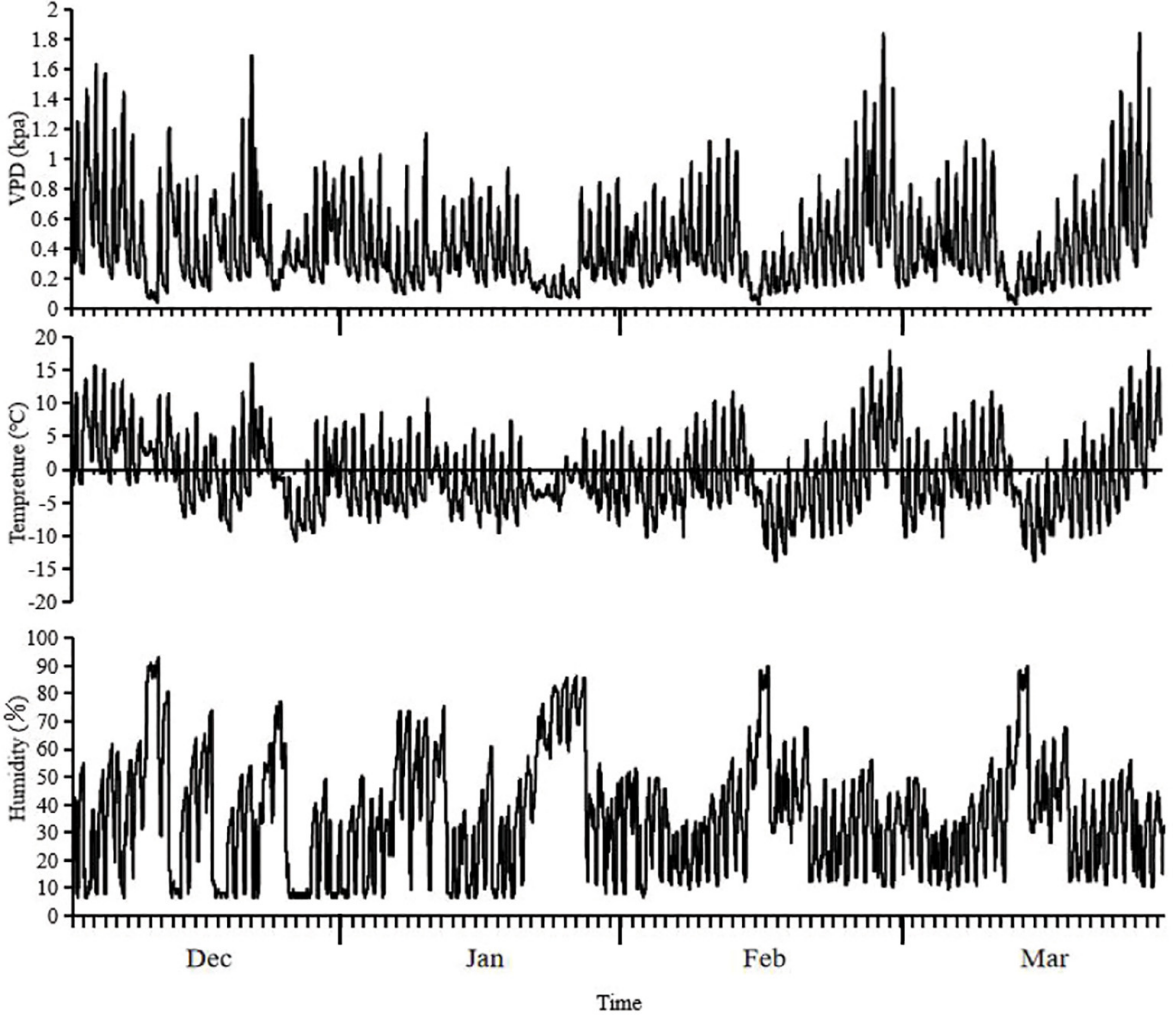
Changes of VPD, temperature and humidity during the wintering period in the test site.

From December 2021 to March 2022, the average VPD, average temperature, and average humidity at the test site during the four-month wintering period were 0.42 ± 0.28 kPa, -0.87 ± 5.50°C, and 35.99 ± 20.66%, respectively. The highest temperature recorded during the test period was 17.9°C on 27 February, whereas the lowest temperature was -13.8°C on 15 February. The average underground soil temperature was approximately 3.8 ± 2.26°C, and the air temperature remained<0°C for 45 days (**Figure 3**).

### 3.2 Embolization ratio and VCs curve changes

**Figure 4:**
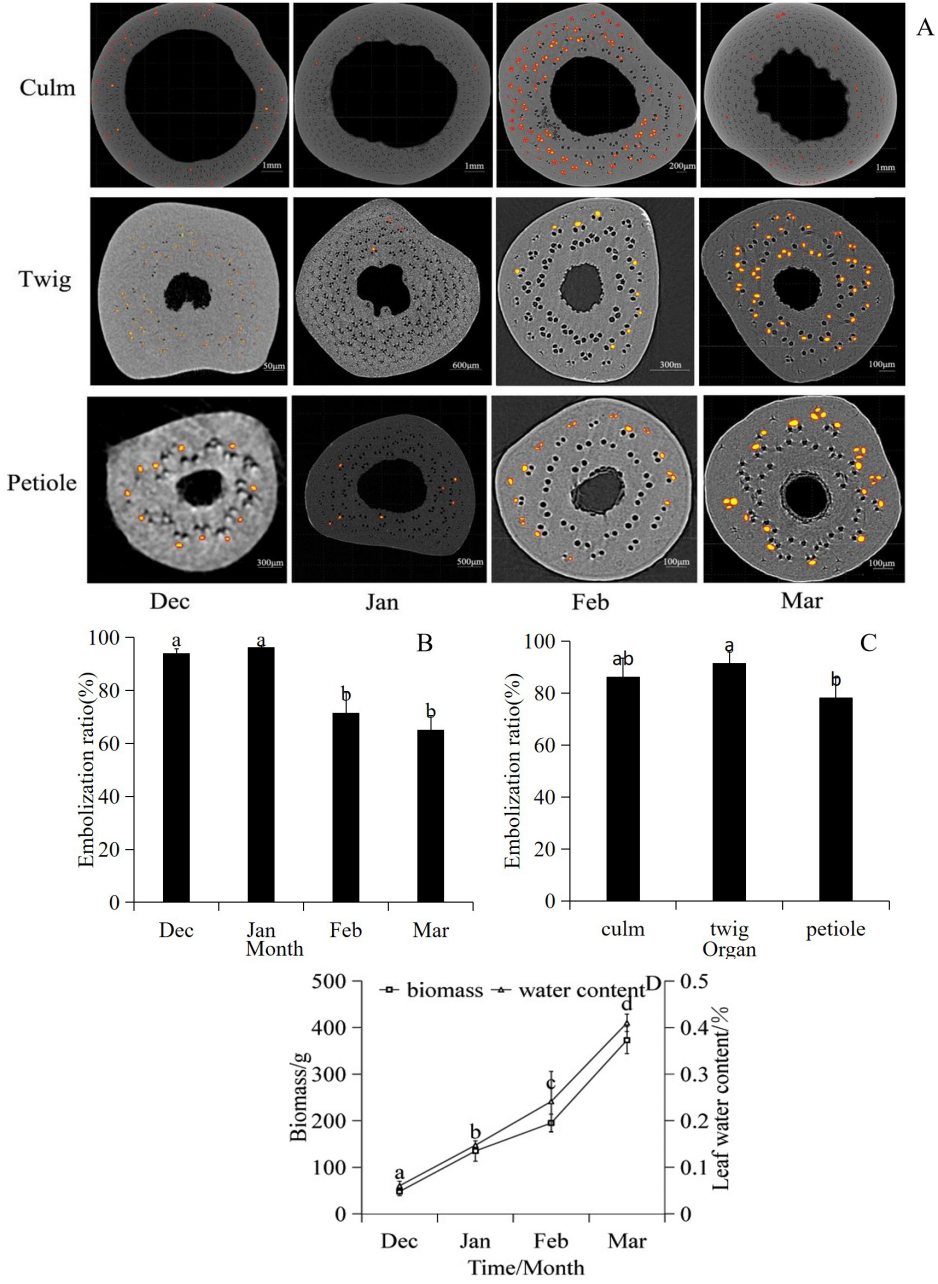
**(A)** CT images of embolization at different part of bamboo organs during the overwintering period. Bright yellow marks represent catheters with water, and no colored catheters represent embolism. **(B)** Embolization ratio varies monthly during wintering period. **(C)** Changes of embolization ratio in different parts of bamboo organs during wintering period. **(D)** Changes of leaf biomass and leaf water content in winter. In **(A–D)** experiments, n=10, three repetitions of each experiment.

From December 2021 to March 2022, significant fluctuations were observed in the culm embolization ratio. The degree of embolism was relatively high during the winter (December to January) and significantly decreased during the spring (February to March). Notably, the ratio of petiole embolisms was consistently lower than that of twigs at each time point (*P*< 0.05). The maximum embolization ratio recorded in January was 99.44%, whereas the minimum petiole embolization ratio observed in March was 54% (**Figures 4A–C**). Concurrently, leaf accumulation increased gradually over time, accompanied by a corresponding increase in the leaf water content (**Figure 4D**).

**Figure 5:**
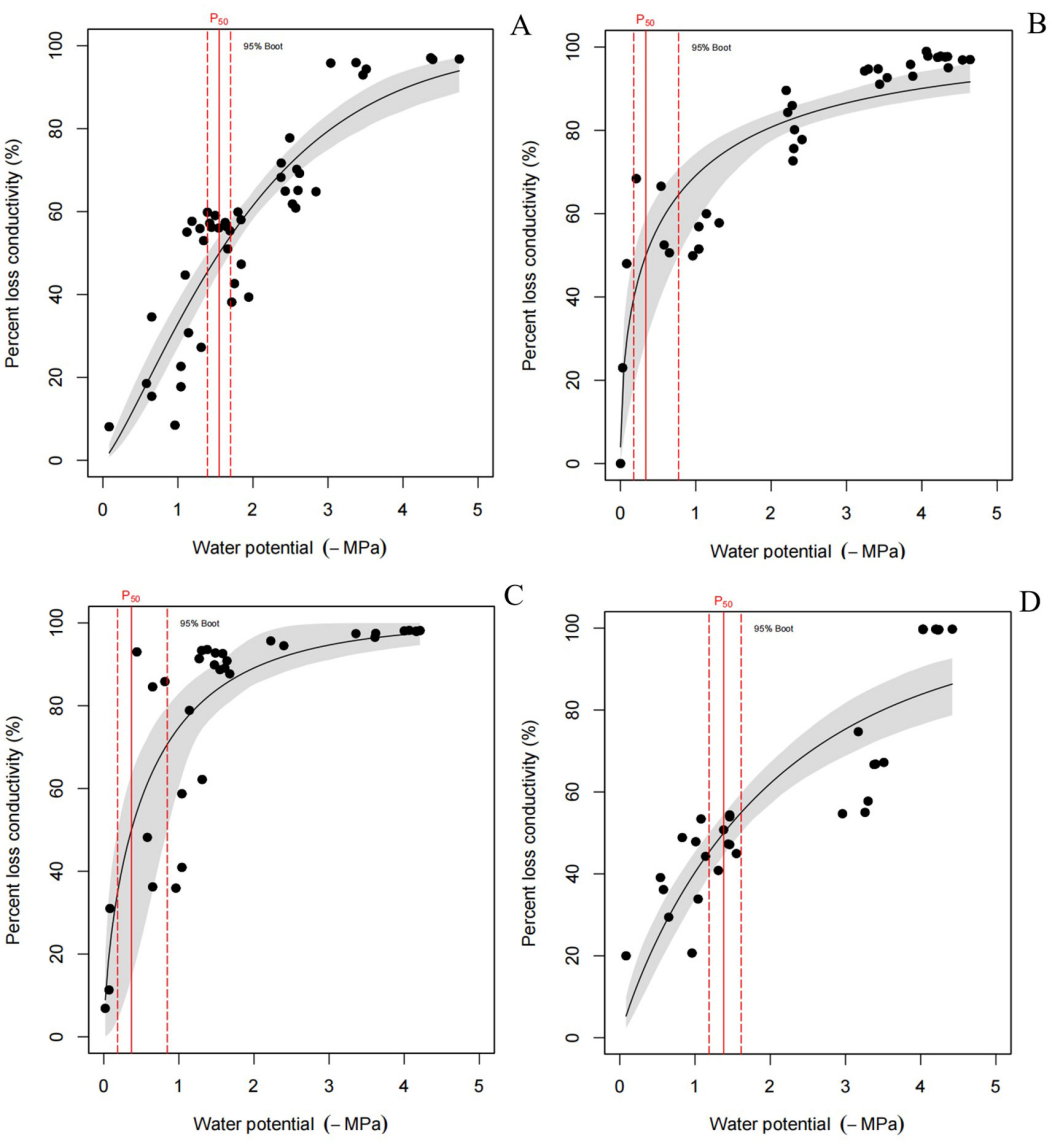
**(A)** Culm embolization VCs curve in December, n=10, three repetitions of each experiment; **(B)** Culm embolization VCs curve in January, n=10, three repetitions of each experiment; **(C)** Culm embolization VCs curve in February, n=10, three repetitions of each experiment; **(D)** Culm embolization VCs curve in March, n=15, three repetitions of each experiment. Different letters indicated significant difference (p<0.05).

There is a direct relationship between xylem embolization and the xylem water potential. When the water potential falls below a certain threshold, the degree of embolization increases rapidly until it reaches its maximum value. A higher threshold corresponds to a greater vulnerability to xylem embolism. The farther to the right of the vertical segment, the higher the water potential threshold; similarly, the higher the distance along the straight segment, the greater the maximum value of embolization and the increased vulnerability to xylem embolism for the corresponding tree species. As illustrated in **Figure 5**, PLC in culms increased rapidly in January and February, reaching 50% PLC at -0.3 MPa and -0.4 MPa, respectively. This indicated that the rate of water conductivity loss was more pronounced during these months. In addition, the PLC of culms at -1.6 MPa and -1.4 MPa in December and March also reached 50%. In this study, the vulnerability of culms to embolism was the highest in February.

### 3.3 NSCs photosynthetic index changes

**Figure 6:**
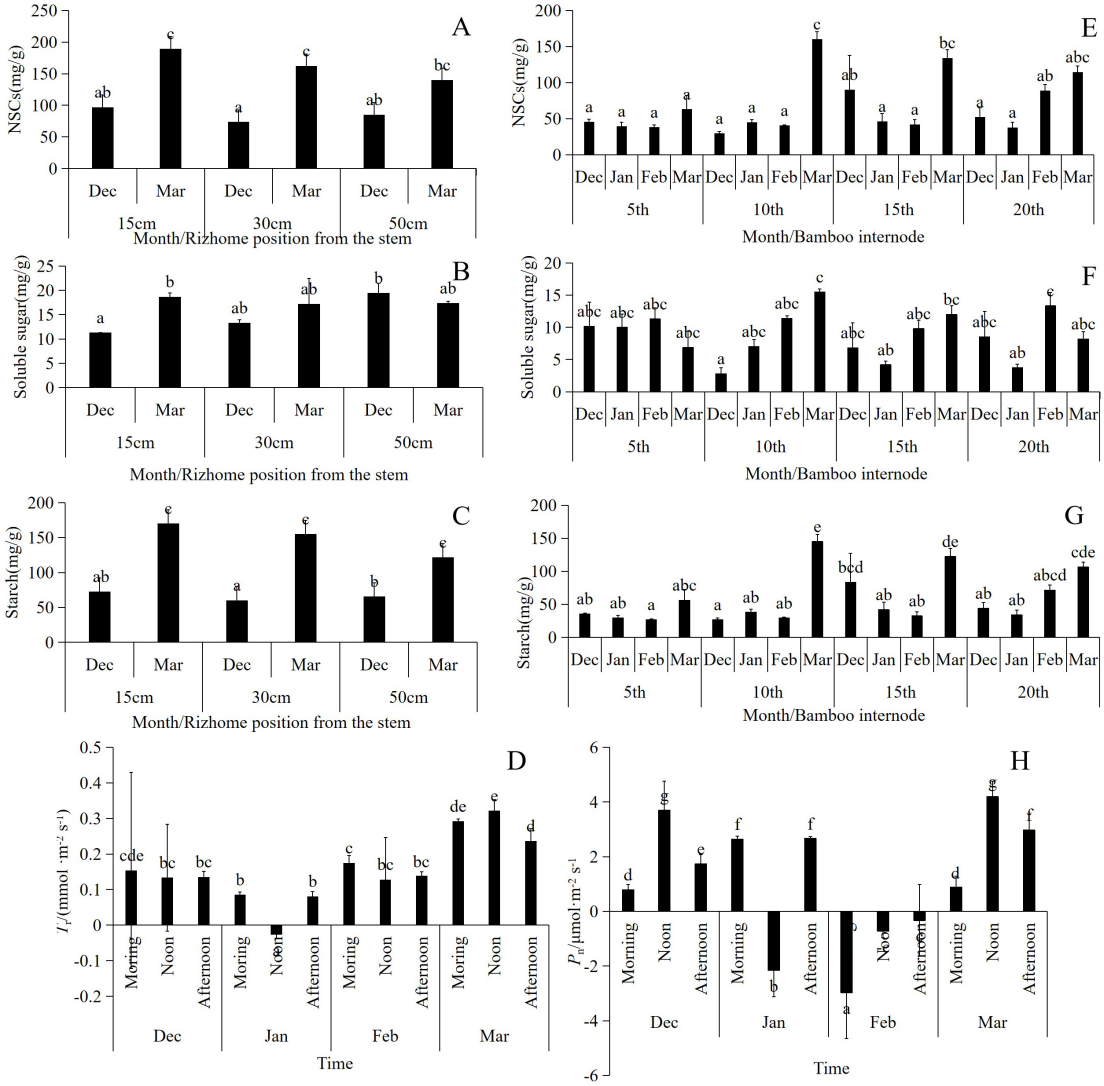
**(A–C)** The changes of NSCs in different parts of rizhome from culm during wintering period, n=12, three repetitions in each experiment; **(E–G)** Changes of NSCs in different segments of bamboo culm during wintering period, n=12, three repetitions in each experiment; **(D, H)** Changes of leaf *T*_r_ and *P*_n_ during the wintering period, n=9, 15 repetitions in each experiment. Different letters indicated significant difference (p<0.05).

The NSCs were lower in January across the various segments. The NSCs in segments 10th and 15th were significantly higher in March than in other months, reaching a maximum value of 160.27 mg/g. NSCs in the underground rhizomes at distance of 15 and 30 cm from the culm were significantly higher in March than in December. In contrast, NSC content in the roots increased at a distance of 50 cm from culm, but this difference was not statistically significant (**Figures 6A, E**).

The range of soluble sugar content in the culms and rhizomes varied from 1.24 to 25.7 mg/g. The soluble sugar content in the rhizomes closer to the culms changed significantly from December to March during the overwintering period, whereas no significant change was observed in the rhizomes farther away. (**Figures 6B, F**).

Starch serves as the primary source of NSCs in *P. vivax* f. *aureocaulis*. The starch content of underground rhizomes exhibited a significant upward trend from winter to spring. Although there was no significant difference in starch content among the different months in the 5th segment of the culm, the starch content in the 10th and 15th segments of the culms increased significantly in March (**Figures 6C, G**). In March, the starch content in the 10th segment reached 169.22 mg/g.

Both *T*_r_ and *P*_n_ exhibited significant diurnal and seasonal variation. Notably, midday *T*_r_ was negative in January (**Figure 6D**). *P*_n_ levels were higher in December and March, and lower in January and February. In both December and March, there were eight days when the midday temperature exceeded 10°C, coinciding with relatively high *P*_n_ levels. Conversely, the *P*_n_ values remained negative throughout February (**Figure 6H**).

**Figure 7:**
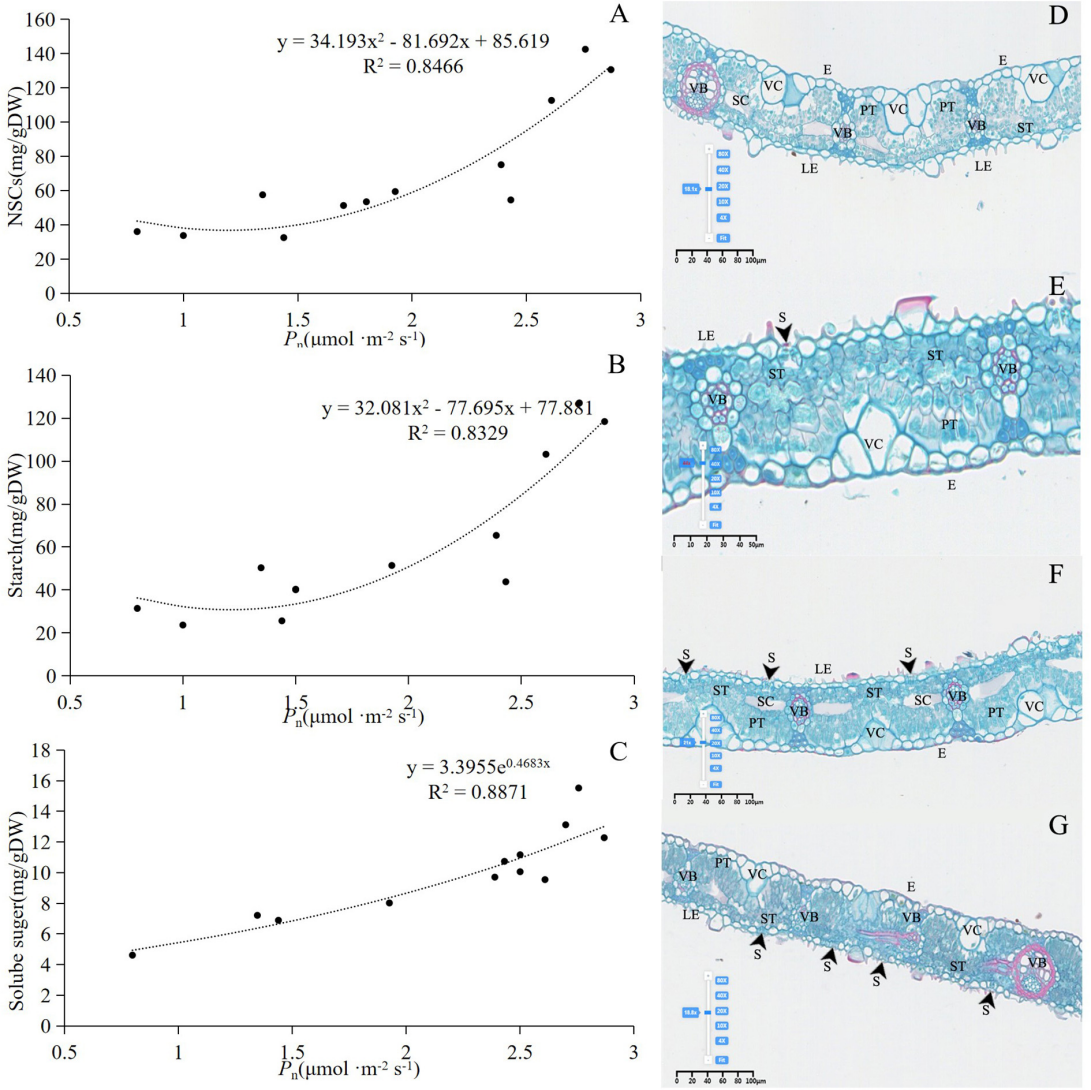
**(A)** Relationship between *P*_n_ and NSCs. **(B)** Relationship between *P*_n_ and starch content. **(C)** Relationship between *P*_n_ and Solube suger. **(D)** Structure of leaves in December. **(E)** Structure of leaves in January. **(F)** Structure of leaves in February. **(G)** Structure of leaves in March. In **(D–G)** experiments, n=9, three repetitions of each experiment. VC, Vesicular cell; S, stomata; PT, Palisade tissue; ST, Spongy tissue; VB, Vascular bundle; SC, Spindle cell; E, Epidermis; LE, Lower Epidermis.

*P*_n_ significantly influenced the changes in NSCs, soluble sugars, and starch in bamboo culms (**Figures 7A–C**). The number of chloroplasts in the bamboo leaves decreased in December. In January, the proportion of spongy tissue increased, whereas the proportions of palisade tissue and spindle cells decreased significantly, indicating decreased gas exchange. In February, the number of spindle cells increased, palisade cells became more distinct from the spongy tissue, and the volume of spongy tissue decreased. Multiple instances of vascular bundle gas ejection were observed in March (**Figures 7D–G**). During winter, the degree of stomatal opening in both the lower and upper parts of the canopy was significantly greater in March than in December, January, and February. In addition, the middle part of the canopy exhibited a significantly higher degree of stomatal opening in January and February than in December and March (**Table 1**).

**Table 1:** Stomatal openness of leaves in winter.

Month	Values	Average number of opened stomatal			Average number of closed stomatal			The ratio of opened stomatals/%			Number of spindle cells		
		Upper crown	Middle crown	Lower crown	Upper crown	Middle crown	Lower crown	Upper crown	Middle crown	Lower crown	Upper crown	Middle crown	Lower crown
Dec	value	11.33c	11.67b	9b	87.33b	81.67b	76.33b	12.64b	14.28b	11.79b	30.33a	21.33b	8.33c
	std	2	3	3	37	14	9				7	11	6
Jan	value	16b	19.33a	12.33b	95.33a	102.67a	81b	16.79b	18.8a	15.19b	16.33b	27b	8c
	std	7	7	4	3	22	26				4	14	6
Feb	value	18.67b	19.67a	9.67b	101a	101.33a	88.33b	18.48b	19.41a	10.95b	25.67a	42a	15b
	std	4	12	1	21	19	35				8	4	5
Mar	value	36a	10b	43a	71b	89b	147a	50.7a	11.24b	29.25a	35a	34a	36a
	std	5	2	5	4	5	5				4	5	4

Different letters in each column indicate significant difference (p<0.05). n=9, three repetitions of each experiment.

### 3.4 Changes in size, morphology, and inclusions of stem cell structures

Although the temperatures were low in December and January, the bundles and cells remained intact (**Figures 2A, B, E, F**). In February, the bundles and cells exhibited signs of fragmentation, accompanied by the presence of invading bodies (**Figures 2C, G**). By March, the bundles and cells returned to their normal state (**Figures 2D, H**). In addition, a significant number of starch granules appeared in the parenchyma cells during March (**Figure 2I**, **Table 2**). Throughout the experiment, the area of the vascular bundles decreased as the number of segments increased, with the area of the aboveground vascular bundles being greater than that of the underground vascular bundles. The average, maximum, and minimum sizes of the vascular bundles in February were smaller than those observed during other periods, indicating that during the winter overwintering phase, vascular bundles contracted to adapt to low winter temperatures (**Figure 2I**).

**Table 2:** Changes of vascular bundle, pitted pore and parenchyma cell structure indexes during overwintering period.

Index	Dec	Jan	Feb	Mar
V_max_/μm^2^	0.05 (0.02)a	0.06 (0.01)a	0.05 (0.01)a	0.08 (0.01)b
V_min_/μm^2^	0.03 (0.01)a	0.02 (0.00) a	0.02 (0.00)a	0.02 (0.01)a
V_ave_/μm^2^	0.05 (0.02)a	0.05 (0.03)a	0.03 (0.00)a	0.06 (0.01)a
VD/number	52.83 (6.85)a	58 (17.88)a	53.33 (21.41)a	54.75 (19.08)a
VP/%	0.40 (0.084)a	0.39 (0.12)a	0.36 (0.08)a	0.43 (0.06)b
N_dp_/number	24.4 (0.98)a	18.75 (6.87)a	17.67 (6.03)a	17.71 (10.57)a
N_pp_/number	30.4 (5.34)a	4 (3.46)c	5.5 (2.08)c	12.93 (11.08)b
D_dpl_/μm	4.65 (0.7)a	1.29 (0.95)b	1.43 (0.84)b	3.39 (0.28)a
D_dps_/μm	2.93 (2)a	0.35 (0.20)c	0.50 (0.27)c	1.65 (0.31)b
D_ppl_/μm	5.35 (0.42)a	0.53 (0.45)c	0.68 (0.50)c	1.59 (0.74)b
D_pps_/μm	2.46 (0.19)a	0.48 (0.30)b	0.40 (0.27)b	0.85 (0.58)b
WT_pc_/μm	11.07 (1.27)a	2.34 (0.91)b	1.55 (0.73)c	2.99 (0.65)b
A_pcp_/μm^2^	2.90 (0.37)a	0.37 (0.30)c	0.55 (0.40)c	1.02 (0.58)b
A_dp_/μm^2^	7.00 (8.44)a	0.86 (0.70)b	1.19 (0.47)b	2.27 (1.91)b

V_max_, maximum vascular bundle area; V_min_, minimum vascular bundle area; V_ave_, average vascular bundle area; VD, vessel density; VP, percentage of sapwood occupied by vessels; N_dp,_ the number of pit holes in the duct; N_pp,_ the number of pit holes in the parenchyma cell; D_dpl,_ the diameter of the long axis of the pit in the duct; D_dps,_ the diameter of the short axis of the pit in the duct; D_ppl,_ the diameter of the long axis of the pit in the parenchyma cell; D_pps,_ the diameter of the short axis of the pit in the parenchyma cell; WT_pc_, xylem parenchyma cell wall thickness; A_pcp,_ parenchyma cell pit chamber area; A_dp,_ duct pit chamber area. Values are means SE. N=12, three repetitions of each experiment. Different letters in each column indicate significant difference (p<0.05).

As shown in **Table 1**, the diameter of the short axis of the pit in the duct (D_dps_), the diameter of the long axis of the pit in the duct (D_dpl_), the number of pit holes in the parenchyma cell (N_pp_), and parenchyma cell pit chamber area (A_pcp_) were lower in January and February than in December and March, respectively. There were no significant differences in the vessel density (VD) or the number of pit holes in the duct (N_dp_) during the overwintering period. The maximum vascular bundle area (V_max_) in March was significantly greater than that in the other months (**Table 2**).

## 4 Discussion

### 4.1 Formation of embolism

Embolism increased significantly during winter due to persistent embolism caused by freeze-thaw cycles (Nardini and Salleo, 2000). In this study, the average temperature was -0.87°C, with the lowest recorded temperature of -10.6°C occurring in December 2021, specifically dropping to -10.6°C on 25 December. This indicated a rapid decrease to a higher level during this period. The average temperatures in January and February 2022 were -1.02°C and -2.06°C, with minimum temperatures of -9.6°C and -13.8°C, respectively. These data indicate a persistently low-temperature environment. However, the average temperature in March remained low at -1.02°C, with the lowest temperature dropping to -13.8°C on 15 March, which adversely affected embolism repair. Although the presence of soluble substances in the plant reduced the freezing point (**Figures 6B, F**), the minimum temperature was significantly lower than the freezing point (**Figure 3B**), preventing the restoration of water potential at night and hindering embolism repair.

In this study, the embolization ratio was in the following order: twig > culm > petiole. This variation was attributed to the high sensitivity of bamboo xylem ducts to cavitation induced by abiotic stress. Even under optimal soil moisture conditions and normal photosynthetic gas exchange, embolization can occur in xylem ducts, leading to water transport failure (Cochard et al., 1994). Interestingly, bamboo thrives under conditions of severe culm embolism, suggesting low water requirements during winter months. However, an increase in the number of dead leaves was noted as winter progressed, coinciding with an increase in leaf biomass (**Figure 4D**). This observation aligns with previous studies, indicating that plants can minimize the risk of embolism by sacrificing the smallest and most peripheral expendable parts, thereby protecting organs with high carbohydrate accumulation (Zimmermann, 1983).

### 4.2 Embolic recovery process and NSCs relieve xylem embolism in winter

To adapt to low temperatures, plants actively accumulate carbohydrates, reduce osmotic potential and freezing points, enhance the osmotic regulation capacity of their cells, prevent freezing induced by low temperatures and mitigate xylem embolism caused by freezing and thawing. In addition, they decrease the annual frequency and duration of freeze-thaw cycles (Charrier et al., 2015; Lintunen et al., 2017).

In December, NSCs and starch contents were lower in various parts of the rhizome and different segments of the culm. The soluble sugar content was higher in rhizomes located farther from the culm, and no significant changes were observed in the different segments of the culm (**Figures 6A–C, E–G**). This observation suggests that the freezing point can be lowered by maintaining non-structural carbon in living cells and xylem vascular bundles, thereby reducing the risk of freezing (Charrier et al., 2015). Although the chlorophyll content and photosynthetic capacity of the leaves decreased (**Figure 7D**), the anatomical structure of the cells remained intact, and starch granules were still observed (**Figures 2A, E**).

NSCs, starch, and soluble sugar contents were lower in various internodes in January (**Figures 6E–G**). However, the photosynthetic rate was higher when temperatures were relatively elevated (**Figure 6H**), indicating that increased photosynthetic rates supplied the resources necessary for the production of antifreeze substances, thereby enhancing cold resistance (Hamilton et al., 2016). Concurrently, the proportion of spongy tissue increased, improving aeration capacity (**Figure 7E**). Loosely arranged and irregularly shaped spongy tissue cells can enhance light scattering, extend the transmission path of light within the mesophyll, and maximize the absorption of limited available irradiance. Photosynthetic products are transported from the leaves to other organs for energy metabolism, osmotic regulation, and synthesis of defense compounds (Hartmann and Trumbore, 2016).

There were no significant differences in the levels of NSCs and starch between February and January, and soluble sugar concentrations were significantly increased in the 20th culm (**Figures 6E–G**). Photosynthesis remained negative throughout the day in February (**Figure 6H**), and soluble sugar levels were significantly positively correlated with *P*_n_ (**Figure 7C**). This correlation suggests that osmotic substances, such as hexose, are produced through photosynthesis and starch granule hydrolysis under prolonged low temperatures, which increases the concentration of cell fluids. Elevated soluble sugar concentrations play a crucial role in cold hardening, helping plants cope with low-temperature stress (Sicher, 2011; Sitnicka and Orzechowski, 2014).

In March, despite the low temperatures, the NSCs and starch content in various internodes of the culm and different positions of the rhizome significantly increased (**Figures 6A, C, E, G**). The parenchyma cells were filled with starch granules (**Figures 2D, H**), and *P*_n_ also showed a significant increase (**Figure 6H**). The soluble sugar content in the 10th section was significantly higher in March than in other months; however, the soluble sugar levels in the 5th, 15th, and 20th sections in March did not differ significantly from those in other months (**Figure 6F**). In addition, leaf structure, photosynthetic conversion capacity, and soluble sugar content were enhanced (**Figure 7G**). In contrast, embolization ratios of the culm, twig, and petiole decreased (**Figure 4B**). Culm embolism vulnerability was lower in the spring of the following year (**Figure 5D**), and high water conductivity facilitated more efficient water transport to the bamboo leaves. This resulted in increased stomatal opening (**Table 1**) and higher transpiration and photosynthetic rates (**Figures 6D, H**, **7G**) (Farquhar and Sharkey, 1982; Franks, 2006). The elevated concentration of soluble sugars causes sugar to transfer from the symplast to the exoplasm, specifically within the vessel, leading to increased culm pressure and the reflow of the embolized vessel (Mayr et al., 2014). Numerous studies have demonstrated a strong correlation between xylem hydraulic efficiency and C assimilation ability across various tree species (Cowan, 1982; Bacelar et al., 2007; Brodribb et al., 2007), a finding confirmed in this study.

### 4.3 Cell structure influences embolic repair

The average, maximum, and minimum vascular bundles in February were smaller than those in other months. This observation indicated that the vascular bundles contracted during winter to acclimate to lower temperatures. During this period, the vascular bundles and cells exhibited signs of fragmentation in response to the invading bodies. In December and March, the culm ducts were larger (**Table 2**), which facilitated starch storage; however, they also had a greater tube wall surface area, making them more susceptible to developing larger pores or cracks in the pitted membrane or having more imperfect cell walls, which could lead to embolization.

The vascular bundle area of bamboo exhibited a significant increase in March (**Table 2**), and starch granules were observed within parenchyma cells (**Figures 2D, H**). Concurrently, the rate of loss of culm water conductivity and vulnerability decreased (**Figure 5D**). This suggests that a larger vessel diameter (**Table 2**) enhances vessel porosity, thereby increasing the hydraulic transport capacity (Fichot et al., 2009). This improvement in efficiency facilitates water transport from the roots to the leaves (Yin et al., 2018), which, in turn, enhances the photosynthetic rate and C uptake (Bacelar et al., 2007; Franks, 2006). In addition, vascular bundle walls were thicker in December and March (**Table 2**). A thicker vascular bundle wall can strengthen the vascular bundle, prevent deformation, and protect against pit-membrane damage (Hacke et al., 2001). This structural enhancement often helps prevent the formation of embolisms (Wheeler et al., 2005; Jansen et al., 2009; Li et al., 2016; Martina et al., 2021). Previous studies have demonstrated that the presence of pitted pores and parenchyma improves hydraulic connectivity between vascular bundles (Salleo et al., 2004; Sano et al., 2011; Barotto et al., 2016). The area of parenchyma cells and conduit pores increased in December and March (**Table 2**), and there was a significant increase in starch granules (**Figures 2D, H**). This suggests that by enhancing the connections between vascular bundles, the cells surrounding the vascular bundles can function as bridges, facilitating water flow between the vascular bundles and contributing to embolic refilling (Barotto et al., 2016).

## 5 Conclusion

Under repeated freeze-thaw cycles during winter, the embolization ratio of *P. vivax* f*. aureocaulis* increased, while its water conductivity decreased. However, when *P*_n_ remained high, the diameter of the vascular bundles increased, and the activation of NSCs enabled *P. vivax* f*. aureocaulis* to withstand low temperatures. Consequently, the interplay between NSCs, photosynthetic capacity, and anatomical structure collectively influenced the sensitivity of *P. vivax* f*. aureocaulis* to low winter temperatures. This balance enhanced the efficiency and safety of water transport while mitigating the potential risks of embolism.

Fluctuations in the NSC content of culm tissue and xylem sap can occur both daily and seasonally. Specifically, monitoring embolization and NSC dynamics across multiple organs and on finer timescales (i.e., seasonally) during winter will enhance our understanding of bamboo responses to cold conditions. Low temperatures induce starch hydrolysis and alter the concentration of low-temperature-protected sugars such as sucrose. Meanwhile, amino acids, such as proline, arginine, and histidine, accumulate during cold acclimation and serve as important nitrogen reserves that support spring regeneration. However, our data does not provide evidence that these starches are converted to sugars or resist hypothermia through respiratory metabolic consumption. Further studies are required to confirm this hypothesis.

## Data Availability

The original contributions presented in the study are included in the article/supplementary material. Further inquiries can be directed to the corresponding authors.
